# ASIC currents in cultured primate retinal rod and cone cells

**DOI:** 10.1007/s00424-026-03207-7

**Published:** 2026-09-05

**Authors:** Talib Saafir, Tiandong Leng, Koichi Inoue, Zhi-Gang Xiong, Peter MacLeish

**Affiliations:** 1 Department of Neurobiology, Neuroscience Institute, Morehouse School of Medicine, 720 Westview Dr. S.W, Atlanta, GA 30310 USA; 2 Department of Anatomy, Pathology, and Medical Education, Morehouse School of Medicine, 720 Westview Dr. S.W., Atlanta, GA 30310-1495 USA; 3 Department of Anatomy and Cell Biology, Nara Medical University, Nara, 634-8521 Japan

**Keywords:** Monkey rod and cone photoreceptors, Dissociation, ASIC, Electrophysiology, Pharmacology

## Abstract

Acid Sensing Ion Channels (ASICs) are widely expressed in the nervous system and involved in an increasing number of functions such as nociception, mechanoreception, and taste transduction, to name a few. In the CNS, ASICs are involved in synaptic plasticity, learning/memory, and in acidosis-mediated neuronal injury. In retina, ASICs are expressed in rodent, rabbit and monkey retina and pharmacological and gene knockdown studies have indicated changes in the earliest phases of electroretinogram that point to effects on phototransduction. This finding led us to investigate the electrophysiological/pharmacological properties of ASICs in cultured primate rod and cone cells. Patch-clamp recordings showed transient ASIC currents with a threshold pH of ~ 7.0 and pH_0.5_ of 6.15 ± 0.03 and 6.33 ± 0.03 for cultured rod and cone cells, respectively. The currents were almost completely blocked by amiloride and highly sensitive to PcTx-1. Our results for the first time demonstrate the expression of functional ASICs on primate photoreceptors and suggest that ASIC currents in these cells are mediated predominantly by homomeric ASIC1a channels.

## Introduction

ASICs are in the family of epithelial Na^+^ channel-degenerin ion channels. They are amiloride sensitive, activated by a decrease in extracellular pH (pH_o_) and present in both peripheral and central neurons [21, 35, 38]. Four genes encode at least six ASIC subunits that form hetero- and/or homo-trimeric transmembrane channels which are permeable to Na^+^ and Ca^2+^ depending upon subunit composition. In peripheral sensory neurons, activation of ASICs has been linked to nociception, mechanoreception, and taste transduction, to name a few functions [28, 31, 33, 34]. In CNS, ASICs are involved in synaptic plasticity, learning/memory, and fear conditioning [11, 36, 37]. In pathological conditions, activation of Ca^2+^ permeable ASIC1a channels has been shown to play an important role in neuronal cell death associated with various neurological disorders such as brain ischemia [40], multiple sclerosis [16], and trauma [43].

Previous studies using the electroretinogram (ERG) showed that inhibiting ASIC1a channels by PcTx-1 or knock-down using antisense oligonucleotides in rats altered the shape of the photopic ERG a- and b-waves [13, 14], suggesting that ASIC1a channels participate in, at least, the phototransduction process. Whether or not these effects involve acidosis remains to be determined but it seems clear that the first step to understanding the role of ASICs in phototransduction is to determine the expression profiles of the different subunits and electrophysiological properties of ASICs in photoreceptor cells. We found that both rods and cones from the monkey retina contained ASIC currents that are sensitive to amiloride and PcTx-1. Whether these channels are responsible for the ERG changes seen in ASIC knock-down experiments and in retinas treated with PcTx-1 remains to be determined.

## Materials and methods

### Eye dissection

Monkey eyes were obtained from the Emory Regional Primate Center as part of their tissue distribution program, as described in our previous studies [29]. In brief, eyes were collected from male and female Rhesus macaques, ranging in age from 9 months to 22 years. Eyes were sterilized by 5 s immersions in 70% ethanol followed by 2 rinses with sterile balanced salt solution (BSS) containing (in mM): NaCl 139, KCl 3, HEPES 2, Na pyruvate 1, MgCl_2_ 0.5, MgSO_4_ 0.5, NaH_2_PO_4_ 0.5, CaCl_2_ 1.8, NaHCO_3_ 1, Choline Cl 0.1, Phenol red 0.02, Glucose 16. The anterior portion of the eye globe containing the iris and cornea was removed, and the vitreous extracted from the eye cup in BSS. The retinae were isolated from the eyecup, dissected into pieces measuring approximately 3–5 mm per side and maintained in BSS. Retinal pieces were dissociated to yield single cells as described in the following sections.

### Retinal dissociation

Isolated retinal cells were obtained by treatment of retinal pieces with papain following methods of Lam [22] and MacLeish et al. [26]. Briefly, retinal pieces were incubated at 37˚C for 30–40 min, with gentle rocking, in a solution containing (in mM): NaCl 114, NaHCO_3_ 25, Na Pyruvate 1, KCl 3, NaH_2_PO_4_ 0.5, CaCl_2_ 0.5, Phenol red 0.02 combined with 7–10 units/ml of papain (Worthington; 3126), 2.7 mM DL-cysteine hydrochloride (Sigma, C-9768), and 16 mM glucose. The enzyme was removed by rinsing 3 times with BSS. Isolated cells were obtained via trituration of treated retina in BSS containing an additional 16 mM glucose using a glass pipette. Cells were seeded onto treated glass coverslips attached to modified 35 mm plastic dishes (Mattek P35G-0-14-C). Cells were maintained at 37˚C in a 5% CO_2_ atmosphere incubator until used.

### Dish preparation

Cells were grown on glass coverslips coated with antibody 9B5 [27], which supported cell adhesion. First, 35 mm glass bottom dishes (Mattek P35G-0-14-C) were incubated with goat anti-mouse affinity purified IgG (GαM, 0.1 mg/ml) in BSS creating the first antibody layer. After room-temperature incubation (> 1 h), the dishes were rinsed with BSS to remove unbound antibody, then incubated (> 1 h) at 4˚C with monoclonal mouse antibody 9B5. The dishes were then rinsed (3x) with BSS and the cells were seeded onto the coverslips at a density of approximately 1 × 10^6^ per dish. Cells settled undisturbed for 20 min followed by the introduction of equilibrated culture medium. The presence of immobilized antibody 9B5 provided an adhesive substrate for the dissociated monkey retinal cells.

### Cell culture

Retinal cells were maintained in a humidified 5% CO_2,_ incubator at 37˚C in serum-free growth medium consisting of 1–10% L-15 (Sigma L-5520, a medium with high concentrations of amino acids) and 90–99% of a salt solution containing (in mM): NaCl 114, HEPES 2, Na Pyruvate 1, MgCl_2_ 0.5, MgSO_4_ 0.5, NaH_2_PO_4_ 0.5, CaCl_2_ 1.8, KCl 3, NaHCO_3_ 25, Choline Cl 0.1, Phenol red 0.02, Glucose 16. The medium was supplemented with gentamicin and 20 µg/ml bovine serum albumin (BSA). Cells were fed twice a week by replacing 1 ml of the medium with fresh medium. Unless stated otherwise (e.g., data in Fig. 5), cells were used for patch-clamp recordings from day 4 to day 7 in culture.

### Immunocytochemistry

Retinal cells were fixed for 10 min with 4% formaldehyde in phosphate-buffered saline (PBS) and then rinsed and stored at 4 °C in PBS until immuno-processing. Cells were processed for immunocytochemistry by blocking with normal goat serum and permeabilized using 1% Triton X-100 in PBS for 1 h at room temperature. To identify rod and cone cells, we used cell-type specific primary antibodies followed by fluorescent secondary antibody labelling. For rods we used mouse anti-body 4D2 [18] and for cones we used mouse monoclonal antibody, 7G6 [37]. Cells were incubated with primary antibodies for 1 h at room temperature. Cells were rinsed 3 times with PBS and then goat anti-mouse Alexa-Fluor 594 or Alexa-Fluor 488 secondary antibody (Vector) was added for 1 h at room temperature. After rinsing with PBS, cells were mounted with Vectashield containing DAPI nuclear stain, viewed with Zeiss Axioskop fluorescence microscopy, and imaged using Zeiss imaging software.

### Electrophysiology

Electrophysiological recordings were performed in the whole-cell mode of the patch-clamp technique [17] using Clampex 9.2 software (Axon), as described in our previous studies [40]. A holding potential of -70 mV was applied unless indicated otherwise. Cells were viewed using an Axiovert (Zeiss) or Nikon TE2000 phase contrast microscope.

Cells were under constant superfusion with pH 7.4 extracellular fluid (ECF) containing (in mM): NaCl 140, KCl 5.4, CaCl_2_ 2, MgCl_2_ 1, Glucose 10, HEPES 20, at room temperature. A multibarrel fast perfusion system (SF-77B, Warner Instruments) was used to achieve rapid changes of extracellular solutions.

Patch-clamp pipettes were pulled on a Sutter P-97 or Narishige PP-830 puller to a resistance of 3–5 MΩ. Internal solution (IS) contained (in mM): CsF 140, HEPES 10, EGTA 11, TEA 2, CaCl_2_ 1, MgCl_2_ 2, MgATP 4 (300 osm, pH 7.3), or KCl 108, NaCl 10, HEPES 10, EGTA 0.05, MgCl_2_ 5, and ATP 1.

Cones had a mean capacitance of 6.97 ± 0.21 pF (*n* = 35); rods had a mean capacitance of 2.48 ± 0.14 pF (*n* = 35); mean access resistance was 5.41 ± 0.49 MΩ (*n* = 23). Recordings with an access resistance > 10 MΩ were not included in data analysis.

### Stock solutions of pharmacological reagents

PcTx-1 (Psalmotoxin-1; Peptides International), 20 µM in water; Amiloride, 100 mM in DMSO; Zinc chloride, 100 mM in water; Cesium chloride, 500 mM in water. Stock solutions were diluted to the desired concentrations in ECF with added bovine serum albumin (BSA 0.01%).

### Data analysis

Data were expressed as mean ± standard error. Student’s t test and ANOVA were used for statistical analysis where appropriate. A p value < 0.05 is considered statistically significant. The current responses were sorted, statistically analyzed, and graphed using Excel, SigmaPlot, and GraphPad software.

## Results

### ASIC current in monkey rod cells

**Fig. 1:**
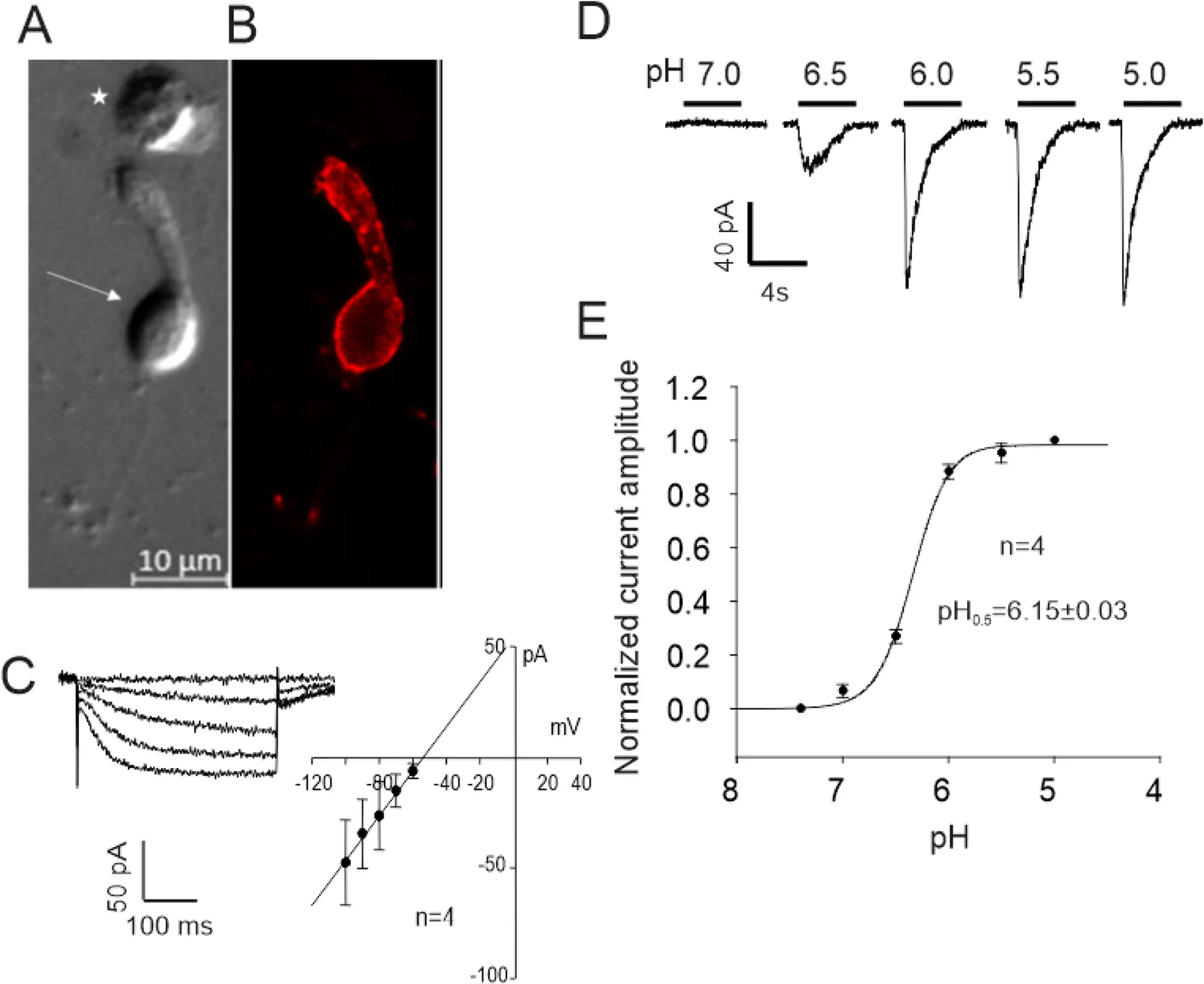
ASIC current in monkey rod cells. (**A**) DIC image of a solitary monkey rod cell (arrow) and unidentified round cell (star). (**B**) Immunolabelling of monkey rod cell with rhodopsin antibody 4D2 and visualized with secondary antibody conjugated to Alexa-Fluor 594 (red). (**C**) Expression of I_h_ in response to voltage steps from − 60 mV to -100 mV in increments of -10 mV. (**D**) ASIC currents at different pH values. (**E**) Dose-response curve showing a pH_0.5_ of 6.15 ± 0.03. Holding potential= -60 mV for C and − 70 mV for D

Rods in low-density culture were identified by their characteristic morphology, and the presence of I_h_ [2, 20], a non-selective cation current mediated by hyperpolarization-activated cyclic nucleotide-gated (HCN) channels that has been shown to exist in photoreceptors. In separate experiments, we used the labeling with the rhodopsin-specific antibody 4D2 [18] to identify rods. The outer segments of isolated monkey rods degenerated within hours in culture but the remainder of the cell consisting of the inner segment, soma, and frequently the rod spherule attached to the soma via a thin process, survived for weeks. Figure 1 A shows the typical morphology of a rod cell and its rhodopsin expression after 7 days in culture. In the absence of an outer segment, rhodopsin is easily observed in the plasma membrane of the soma and inner segment (Fig. 1B), as previously reported [23, 32]. Another criterion of rod cells is the presence of I_h_ as previously reported [19] and shown in Fig. 1C. I_h_ was clearly expressed at potentials of -70 mV and below and showed a prominent tail when the potential was returned to the holding potential (-60 mV). The tail currents deactivated over a time of 100–200 msec.

Using patch-clamp recordings, we found that roughly half of the rods responded to decreases in extracellular pH. Of a population of 37 rods, 21 showed a transient inward current when the pH was lowered from 7.4 to one as low as 4.0. An example is shown in Fig. 1D. The current showed prominent inactivation in the presence of low pH.

Next, we constructed the dose-response relationship of ASIC currents in rod cells. As shown in Fig.  1E, the threshold pH for current activation was ~ 7.0, the pH for maximal activation was ~ 5.5, and the pH_0.5_ was 6.15 ± 0.03 (*n* = 4).

**Fig. 2:**
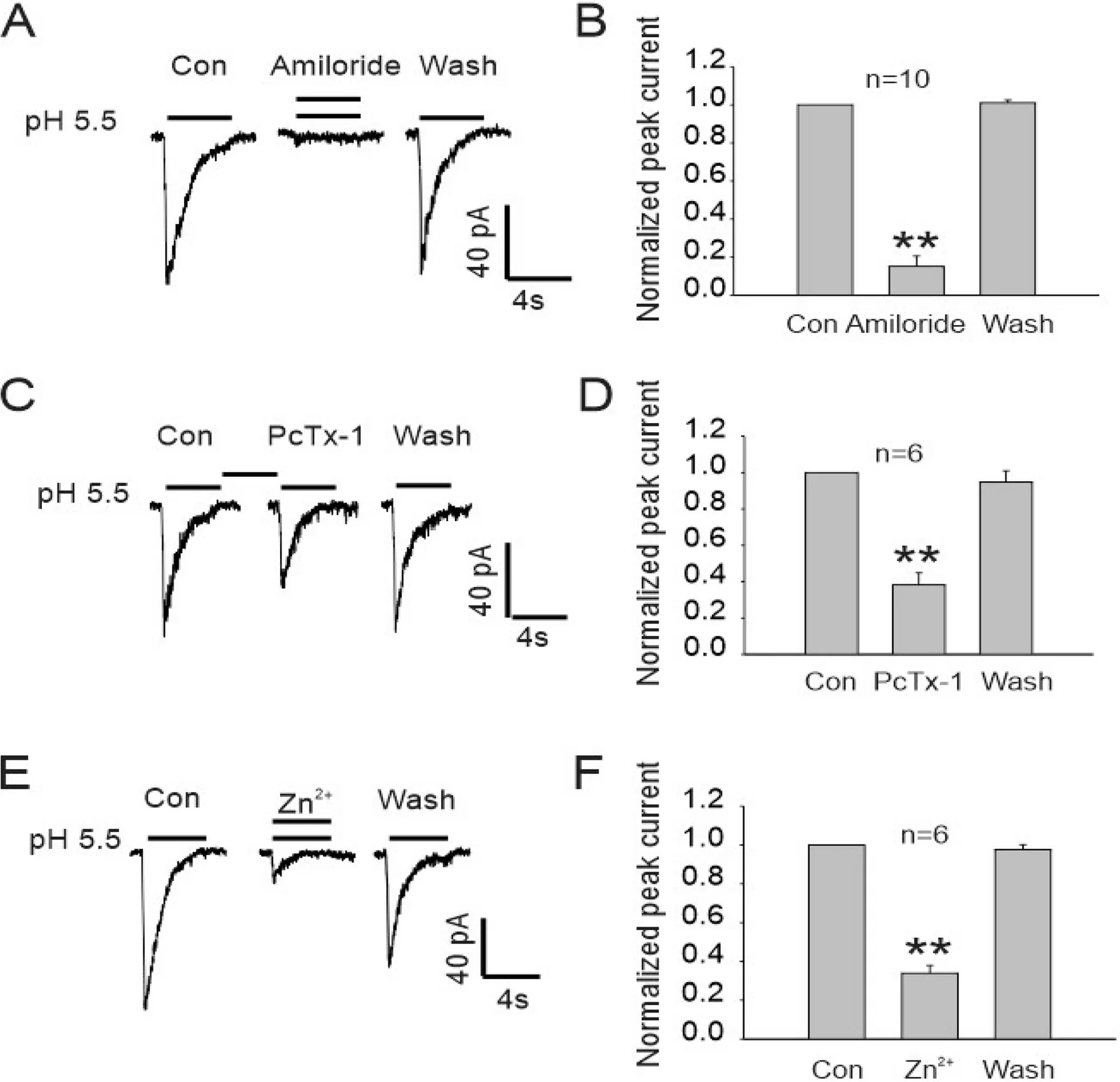
Pharmacology of ASIC currents in monkey rod cells. (**A**) ASIC current activated at pH 5.5 was reversibly suppressed by amiloride (100 µM). (**B**) Amiloride reversibly blocked ASIC currents in rod cells by an average of 84.7% (*n* = 10). (**C**) ASIC current activated at pH 5.5 was reversibly suppressed by PcTx-1 (20 nM). (**D**) PcTx-1 blocked ASICs currents in rod cells by an average of 61.7% (*n* = 6). **E**-**F**) Zinc (300 µM) blocked ASIC currents in rod cells by 66% (*n* = 6). Holding potential= -70 mV

We then determined the pharmacological profiles of ASICs in rods. ASIC currents were reversibly blocked by co-application of amiloride (100 µM), the commonly used non-selective blocker of ASICs (Fig.  2A). The currents were also reversibly suppressed by PcTx-1 (20 nM), a specific inhibitor of homomeric ASIC1a and/or heteromeric ASIC1a/2b channels [12, 30] (Fig. 2C). The average block of peak ASIC currents in rod cells by amiloride was 84.78% (*n* = 10) (Fig. 2B). PcTx-1 reduced the peak amplitude of ASIC currents by an average of 61.74% (*n* = 6) (Fig. 2D), suggesting that ASIC currents in rod cells are primarily mediated by ASIC1a containing channels.

To further determine the subunit composition of ASICs in rod cells, we tested the effect of zinc. Previous studies showed that extracellular zinc inhibits ASIC1a-containing channels with high affinity [8] while a high concentration of zinc (e.g., 100–300 µM) potentiates ASIC2a containing channels [4]. As shown in Fig. 2E, the application of 300 µM zinc did not potentiate but suppressed the rod ASIC currents by 66% (*n* = 6) (Fig. 2F), further suggesting that the ASIC currents in monkey rod cells were predominantly mediated by ASIC1a containing channels.

### ASIC current in monkey cone cells

**Fig. 3:**
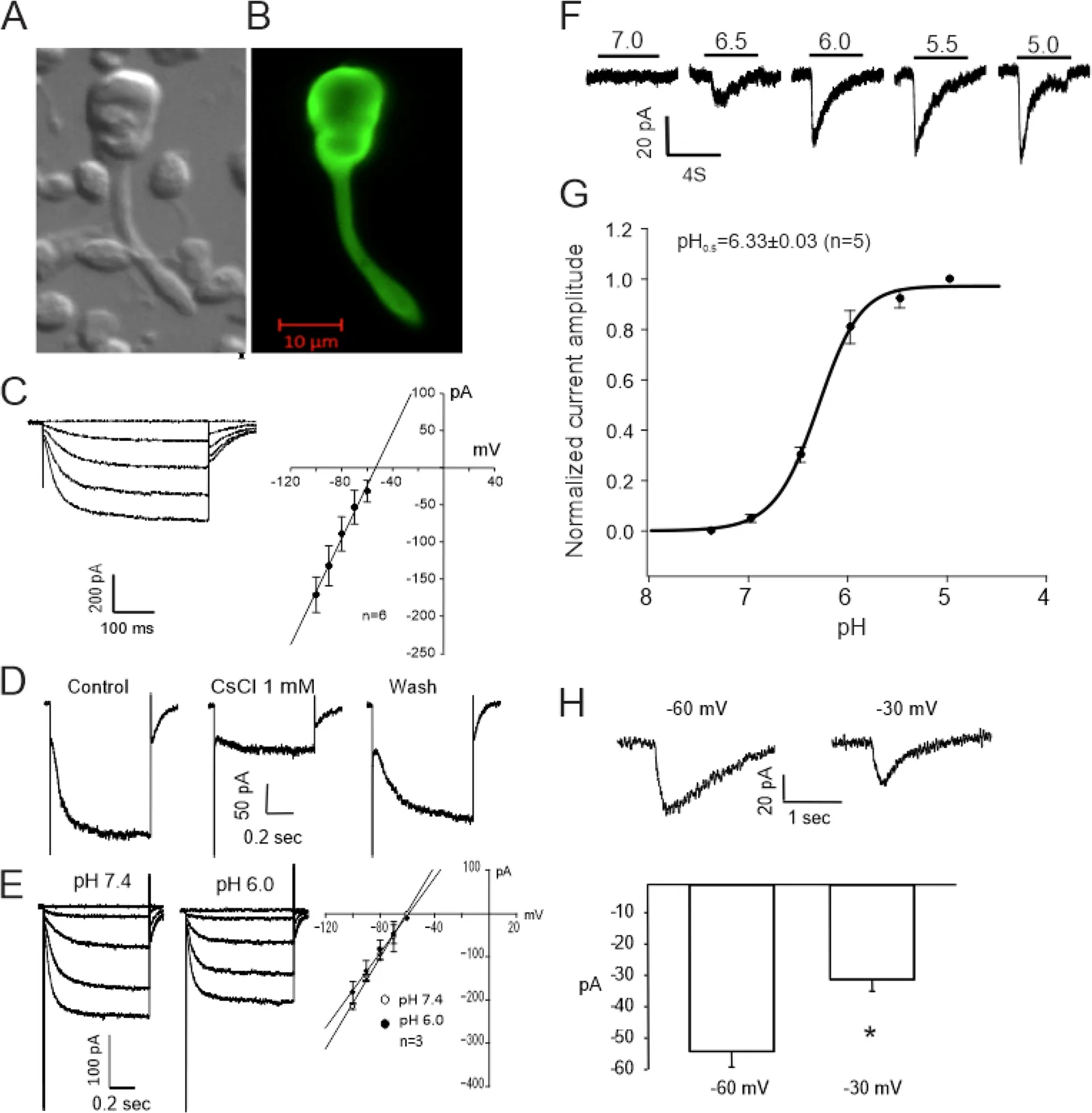
ASIC currents in monkey cone cells. (**A**) DIC image of a solitary monkey cone cell. (**B**) Immunolabelling of monkey cone cell with cone-specific antibody 7G6 and visualized with secondary antibody conjugated to Alexa-Fluor 488 (green). (**C**) Expression of I_h_ in response to voltage steps from − 60 mV to -100 mV in increments of -10 mV. (**D**) Suppression of I_h_ by 1 mM CsCl (*n* = 4); voltage step was − 100 mV. (**E**) Effect of pH 6.0 on I_h_; same protocol as in C (*n* = 3). (**F**) ASIC currents at different pH values. (**G**) Dose-response curve showing a pH_0.5_ of 6.33 ± 0.03 (*n* = 5). (**H**) Responses to pH 5.5 at potentials of -60 mV and − 30 mV (*n* = 3, *p* < 0.05). Holding potential = -60 mV for **C**-**E **and − 70 mV for **F**

Cones were identified by their morphology, by the labeling with a cone-specific antibody 7G6 [39] and by the presence of I_h_ as previously reported [3, 19, 41]. Figure 3 A and B show the morphology and labeling with antibody 7G6 in a cone cell that was in culture for 7 days. Another criterion for cone cells is I_h_, which was present in cones as shown in Fig. 3C. I_h_ activated with a characteristic time course at voltages negative to -60 mV and showed prominent tail currents when voltage was returned to -60 mV. The time course of deactivation of the tail current at -60 mV was 100–200 msec. I_h_ was reversibly blocked by 1 mM CsCl (Fig. 3D).

We further examined the effect of pH on I_h_. We observed a slight decrease in the amplitudes of I_h_ and a shift in the reversal potential at pH 6.0 compared to those at pH 7.4 but changes are not statistically significant (*n* = 3) (Fig. 3E).

Like rod cells, roughly half of cones were sensitive to lowering pH. Of a total of 197 cones recorded, 91 showed a transient inward current when pH was lowered from 7.4 to 6.5 or below (Fig. 3F) at a holding potential of -70 mV.

We then determined the dose-response relationship of ASIC currents in cone cells. As shown in Fig. 3G, the threshold pH for current activation was ~ 7.0, the pH for maximal activation was ~ 5.5, and the pH_0.5_ was 6.33 ± 0.03.

Although the possible involvement of ASIC in the light response was not the primary focus of this study, we decided to compare the response of our light-adapted cone cells at a potential reported for cones in darkness with that measured in light. In the intact retina under dark adapted conditions, the resting potential of cone cells is in the vicinity of -40 mV while the membrane potential of cone cells in bright light is in the vicinity of -55 to-60 mV. Figure 3 H shows the response of a cultured cone cell to a solution at pH 5.5 at a holding potential of -60 mV or -30 mV. Cone cells responded to the low pH solution with inward currents at both holding potentials but the response amplitude at -60 mV was larger than that at -30 mV.

**Fig. 4:**
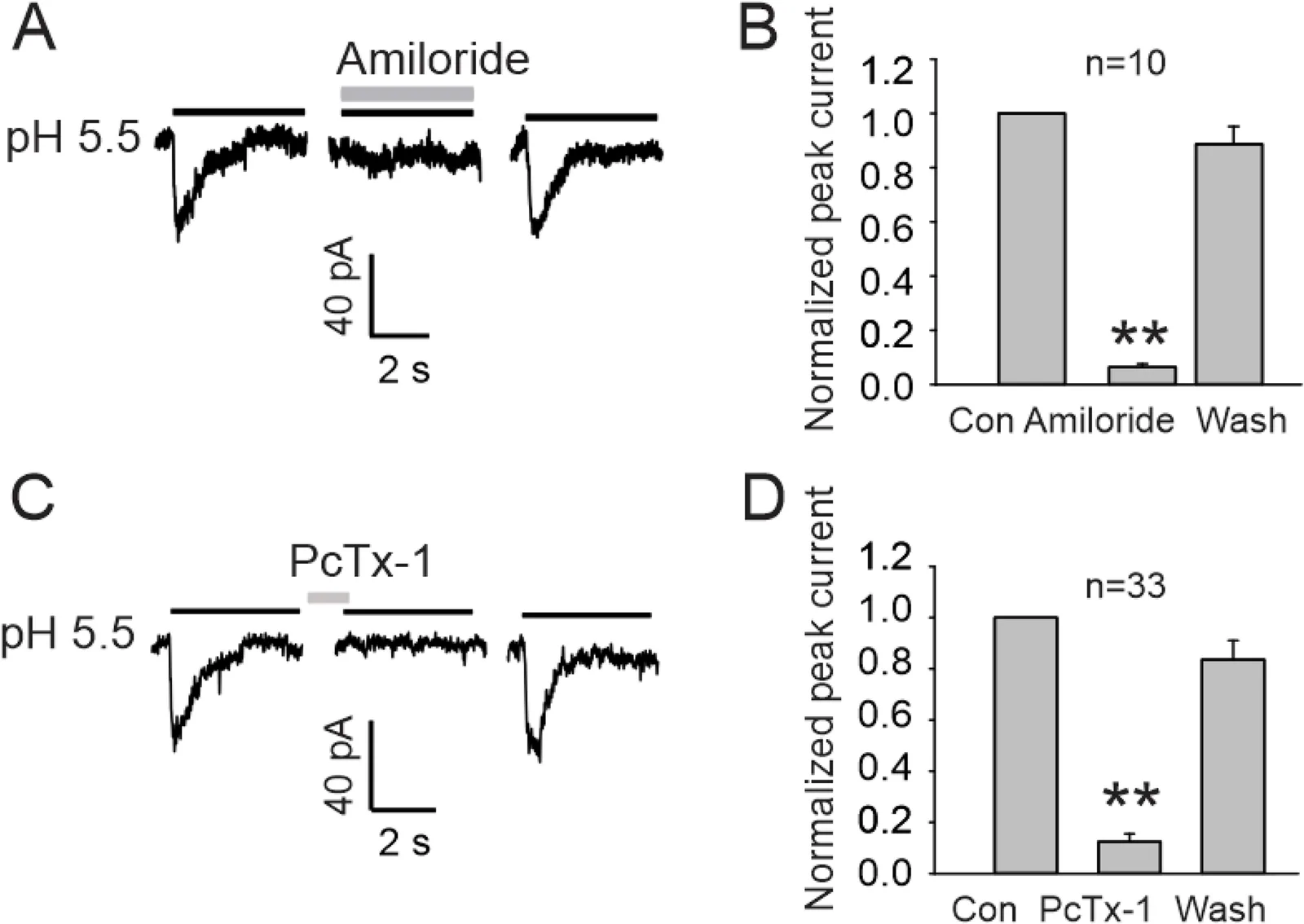
Pharmacology of ASIC currents in monkey cone cells. (**A**) ASIC current was reversibly suppressed by amiloride (100 µM). (**B**) ASIC current was reversibly suppressed by PcTx-1 (20 nM). (**C**) Amiloride blocked ASIC currents in cone cells by an average of 93.6% (n = 10). (**D**) PcTx-1 blocked ASIC currents in cone cells by an average of 87.6% (*n* = 33). Holding Potential=-70 mV

Next, we determined the pharmacological profiles of ASICs in cultured cones. Similar to the findings in rods, ASICs in cones were blocked by amiloride and PcTx-1. The peak amplitudes of ASIC currents in cones decreased by 93.62% in the presence of 100 µM amiloride (*n* = 10, Fig. 4A&B) and 87.63% in the presence of 20 nM PcTx-1 (*n* = 33, Fig. 4C&D). Thus, the ASIC currents in cone cells were also predominantly mediated by ASIC1a-containing channels.

### ASIC current in cones as a function of time in culture

Since about half of the rods and cones did not show detectable ASIC currents on days 4–7 after plating (see above), we were wondering whether this sensitivity profile was determined by the number of days in culture and whether the amplitude of ASIC current also changes with culturing days. We focused on cones and examined the expression of ASIC currents in cones isolated from nine animals starting from day 0 in vitro and going up to day 36 in vitro.

**Fig. 5:**
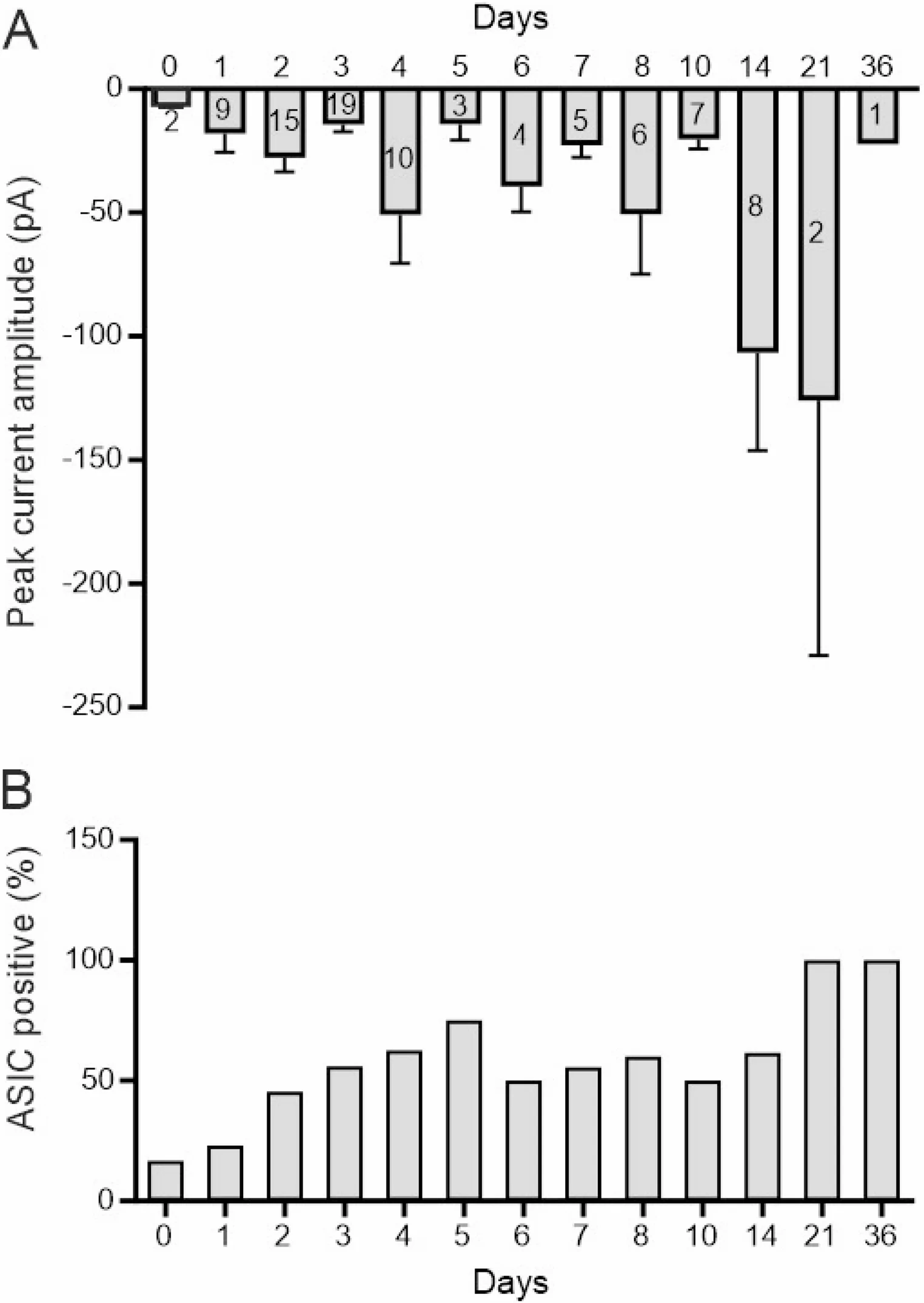
Summary of ASIC responses in cones recorded on different days in culture. On day 0, for example, less than 15% of the cone cells showed detectable ASIC currents with pH drops to as low as 4.0. The percentage of cone cells showing positive ASIC currents gradually increased for the first 5 days, slightly decreased on day 6, and stayed relatively stable up to day 14. The fraction of ASIC-positive cells increased on days 21 and 36. The numbers inside each bar in Fig. 5A represent the sample size of recorded cells for that day. Holding potential = -70 mV

As shown in Fig. 5, less than 15% of the cones showed detectable ASIC currents on day 0 with pH drops to as low as 4.0. The percentage of cones showing detectable ASIC current gradually increased for the first 5 days and stayed relatively stable up to day 14. The fraction of ASIC-positive cones appeared to be increased on days 21 and 36. For the cones that responded to pH drops with detectable currents, the peak amplitude of ASIC currents (activated at pH 6.0) showed large variability ranging from about − 4.9 pA to as much as -354 pA as shown in Fig. 5A. There is a trend that the amplitude of ASIC currents increased with increased days in culture, even though it fluctuated on different days.

## Discussion

In the present study we report the presence and pharmacological properties of ASICs in primate retinal rod and cone cells using patch-clamp techniques. Although the expression of ASICs in retina has been demonstrated in various animals including rats, mice, rabbits and monkeys [13, 14, 25], this is the first report of the functional expression of ASICs in primate retinal rod and cone cells. We contend that the expression of ASICs occurs in situ since it was observed from the earliest times in cell culture.

In a previous study [29], we discovered through the use of RT-PCR that the intact primate retina has strong expression of ASIC1a, 2a, and 4 but weak ASIC3. In addition, we looked for the ASIC expression in cultured retinal cells maintained for different lengths of time *in vitro.* We found that ASIC1a, 2a, and 4 are strongly expressed in cell cultures on day 5. This finding indicates that culturing conditions did not cause a major change in ASIC subunit expression. Our findings in intact retina agree with earlier studies of retinal ASICs in rabbit [7].

In about half of rods and cones studied, transient inward currents were recorded upon bath application of acidic solutions. The amplitude of the currents was pH dependent with a threshold pH of ~ 7.0 and a maximal current recorded at ~ pH 5.5. Detailed dose-response analysis showed a pH_0.5_ of 6.15 ± 0.03 and 6.33 ± 0.03 for rods and cones, respectively. These electrophysiological characteristics of acid activated currents were similar to those of typical ASIC currents recorded in other parts of the nervous system, e.g., mouse cortical and human brain neurons [24, 40]. In addition to amiloride, all responding cones and rods showed high sensitivity to PcTx-1, suggesting the expression of homomeric ASIC1a and/or heteromeric ASIC1a/ASIC2b channels. Since our RT-PCR experiments did not detect clear expression of ASIC2b subunit, our results suggest that ASIC currents in primate photoreceptors are predominantly mediated by homomeric ASIC1a channels.

The presence of ASICs in monkey rods and cones indicates the expression of another family of conductance mechanisms on these cells and expands the number of mechanisms that can modify the photoresponse from 2 first proposed by Baylor et al. [5] to at least 5 shown by several investigators [1, 2, 15].These include voltage-gated conductances, calcium-gated potassium conductance, calcium-gated anion conductance and cyclic GMP gated conductance. In addition, photoreceptor function can be modified by a variety of primary amines such as dopamine, serotonin, melatonin, to name a few, and by neurotransmitters acting through appropriate surface receptors. This array of conductance mechanisms allows for modification of the light response under a wide variety of lighting conditions and for supporting the long-term life of the cells.

In peripheral neurons, the physiological function of ASICs has been linked to nociception, mechanoreception, and taste transduction [21, 38]. In the CNS, studies have suggested that ASICs are involved in synaptic plasticity, learning/memory [36], and protons have been proposed as a neurotransmitter, at least in the amygdala [11]. Limited studies have also suggested their potential functions in retina. Ettaiche et al. [14], for example, showed that silencing ASIC1a alters cone-mediated retinal function, while knockout of ASIC2a increases light sensitivity, light sensitization, and retinal damage [13]. Our current finding that functional ASICs are expressed in monkey retinal photoreceptors sets the groundwork for considering the possibility that the ERG effects arising from knockdown of ASIC might be occurring at the level of the photoreceptors themselves.

As mentioned above, the most direct line of evidence for a role of ASICs in phototransduction comes from the work of Ettaiche et al. [14] which showed changes in the electroretinogram (ERG) waveform when ASIC1a was suppressed by antisense oligonucleotide injection or by the introduction of the ASIC1a specific blocker, PcTx-1, into intact eyes. Injection of either antisense oligonucleotide or PcTx-1 markedly reduced the amplitude of the photic ERG a and b waves. These results strongly suggest that ASIC1a channels are involved in cone function in a manner to be determined. The authors also reported a small but significant effect of the two agents on the a and b waves of the scotopic ERG thereby pointing to a direct effect on rod function as well. The rapid response of the ERG to intravitreal PcTx-1 injection strongly suggests that the ASIC channels responsible for the alteration in light response are likely located on cones. Indeed, Ettaiche et al. [12] reported labeling of cone inner segments with an antibody purported to be specific for ASIC1a channels. Our electrophysiological results confirm the presence of functional ASIC channels on cones and rods and suggest that PcTx-1 might be acting directly on photoreceptors to suppress the light response.

Several explanations can be offered to account for the reduction in the amplitude of the ERG in response to ASIC inhibition or knock-down. First, a general reduction in the retinal extracellular resistance would lead to a reduction in the amplitude of the ERG. A second explanation might be a reduction in the dark current that is suppressed by light. This current is controlled by cyclic GMP so a reduction in the intracellular cyclic GMP could lead to a reduction in the dark current and as a consequence a reduction in the light response. How PcTx-1 or antisense knock-down can cause a reduction in extracellular resistance or of the dark current is not clear. Another question arises from the reduction in the light response due to ASIC1 block or knock-down. A reasonable expectation is that a drop in pH is occurring to allow the effect of PcTx-1 or ASIC1 knock-down to be observed. Changes in retinal pH do occur as a result of circadian rhythms and metabolic activity of retinal cells that is modified by lighting conditions. In the dark, the outer plexiform layer is acidic due to the high release of neurotransmitter from photoreceptors as is the region between the photoreceptors and the retinal pigmented epithelium where the release of lactic acid is prominent [6, 9, 10, 42]. Perhaps the small pH changes observed in the extracellular spaces in the retina are sufficient to produce the observed changes in the ERG.

## Data Availability

No datasets were generated or analysed during the current study.
